# Development of wirelessly-powered, extracranial brain activator (ECBA) in a large animal model for the future non-invasive human neuromodulation

**DOI:** 10.1038/s41598-019-47383-2

**Published:** 2019-07-29

**Authors:** Hyungwoo Lee, Jin San Lee, Yeongu Chung, Woo Ram Chung, Sang Joon Kim, Joon Seong Kang, Sung Min Park, Wonok Kang, Dae Won Seo, Duk L. Na, Young-Min Shon

**Affiliations:** 1 Micro Bio Processor, Computing Platform Lab, Samsung Advanced Institute of Technology, Suwon, Korea; 2 Department of Neurology, Kyung Hee University Hospital, Seoul, Korea; 3 Department of Neurosurgery, Kangbuk Samsung Hospital, Sungkyunkwan University School of Medicine, Seoul, Korea; 4 Department of Neurology, Samsung Medical Center, Sungkyunkwan University School of Medicine, Seoul, Korea; 5 Department of Creative IT Engineering, Pohang University of Science and Technology (POSTECH), Pohang, Republic of Korea

**Keywords:** Neural circuits, Disorders of consciousness

## Abstract

As transcranial electrical stimulation (tES) is an emerging and promising technique for neuromodulation, we developed a novel device; wirelessly-powered, extracranial brain activator (ECBA), which is mounted subcutaneously, and its neuromodulation effect was investigated. The oscillatory changes in electrocorticography (EcoG) were analyzed from two types of stimulation. Two weeks prior to the recording experiment, we underwent surgery for implantation of subdural strips and ECBA module over centroparietal regions of anesthetized beagles. Low-frequency stimulation (LFS) and subsequent high-frequency stimulation (HFS) protocols (600 pulses respectively) were applied. Then, the power changes before and after each stimulation in five different bands were compared. A significantly larger voltage difference with subcutaneous than transcutaneous stimulation measured at EcoG channels indicated a substantial current attenuation between the skin and skull. Compared with the baseline, all subjects showed consistently decreased delta power and increased gamma power after HFS. LFS also induced a similar, but opposite, pattern of power change in four beagles. The results from this study indicate that LFS and HFS with our novel ECBA can consistently and effectively modulate neural activity of the cortex, inducing neural inhibition and facilitation functions, respectively. Future studies are necessary to further ensuring a consistent efficacy and long-term safety.

## Introduction

Recent studies on non-invasive brain stimulation (NBS), including transcranial magnetic stimulation (TMS), transcranial direct current stimulation (tDCS), transcranial, or transorbital alternating current stimulation (tACS), and transcranial random noise stimulation (tRNS) have been proposed to facilitate functional recovery and be applied as possible therapeutic options for neurological and psychiatric diseases including depression, epilepsy, stroke, movement disorders, and dementia^1–5^. In particular, transcranial electrical stimulations (tESs) such as tDCS and tACS are associated with the application of weak electrical currents to improve brain function through distinct physiological mechanisms that vary based on the type of tES^6–8^. However, knowledge regarding how tES modulates electrophysiological activities of the brain is lacking^9^.

tDCS is one of the most widely used tES techniques. tDCS adjusts neuronal excitability by injecting low direct currents to electrodes placed on the surface of the scalp and has been shown to temporarily alter the resting potential of the membranes of affected neurons without causing action potentials^10^. Conversely, tACS uses unidirectional or bidirectional current pulses in rectangular or sinusoidal waves, which modulate ongoing neural oscillations at specific frequencies^7,11^. In the studies with human subjects, ACS-induced after-effects are known to arise from synaptic-level processes^12^; synaptic plasticity refers to the ability of synapses to modify transmission efficacy or strength due to experience over time^13^. Moreover, tACS modulates the neuronal networks in a phase- and frequency-dependent manner, derived from the analysis of human data^14^ or a computational modeling of cortex^15^.

However, the translation of these neuroscientific findings on tES to the human brain is not straightforward because brain morphological findings such as cortical folding and skull thickness are more complex, and minor adverse effects (e.g. skin erythema, pain, or headaches) arise from the current passing through the scalp and the cerebrospinal fluid (CSF) before reaching the cortex^16,17^. The electrical attenuation through human scalp and skull adds more complexity to translate the animal study results to real-world human application. Therefore, the mechanism of tES remains less understood and raises an important issue whether stimulation with weak current can induce significant transcranial CNS effects.

A possible solution to these issues is an extracranial brain stimulator which is mounted subcutaneously and wirelessly-powered such as a magnetic inductive-powered extracranial brain activator (ECBA), which can deliver more efficient currents directly and consistently through the skull in freely moving condition. In addition, ECBA supports various types of alternating current waveforms and is a state-of-the-art stimulating technique selected for its specific neuromodulation.

In this study, a series of proof-of-principle tests with electrocorticography (EcoG) under different stimulation parameters were reported, demonstrating ECBA is appropriate for inducing direct alterations in intrinsic cortical oscillation, thus confirming potential applications in basic or clinical research.

## Materials and Methods

### Animals and surgical implantation of ECBA and subdural electrodes

**Figure 1:**
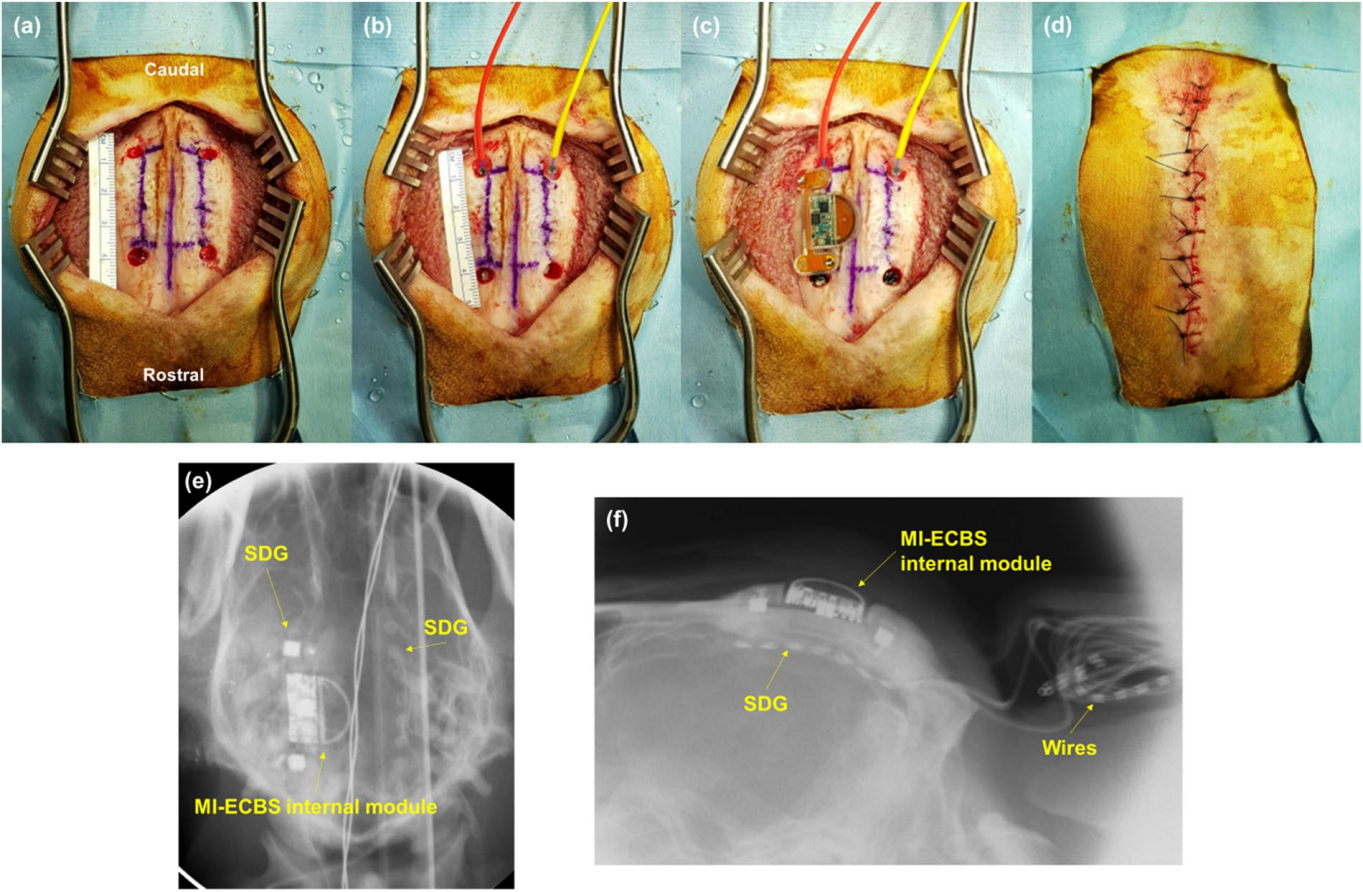
The procedure of implanting SDG electrodes and the ECBA system. (**a**) Four burr holes drilled in the dog’s skull; (**b**) two SDG electrodes inserted into the bilateral cerebral cortex parallel to the midline; (**c**) the ECBA internal module fixed on the right skull of the dog; (**d**) closure of the incision; the location of ECBA with SDG electrodes as seen from (**e**) anteroposterior X-ray view and (**f**) left lateral X-ray view. SDG, subdural grid; ECBA, wirelessly-powered extracranial brain activator.

All surgeries for implanting subdural electrodes and ECBA were performed by one neurosurgeon (Chung Y) in five healthy male beagles. All dogs, weighing 11–13 kg, were initially sedated with Telazol (5 mg/kg i.m.) and xylazine (2 mg/kg i.m.), then intubated with an endotracheal tube and continuously ventilated with isoflurane (1%) in the prone position under a standard stereotactic apparatus. A midline incision, approximately 70 mm-long, and exposure of the cranial landmarks, bregma and inion, was performed to facilitate drilling of four burr holes (5 mm in diameter, 35 mm apart and 10 mm off the midline) using a high-speed drill on each hemisphere (Fig. 1a). Subsequently, two subdural strip electrodes (3 mm contact diameter, interelectrode interval 5 mm, 8 contacts; PMT Corporation, Chanhassen, MN, USA) were inserted parallel to the midline in a caudal-rostral direction (Fig. 1b). The cables (opposite to the electrodes) were rolled up and buried in a subcutaneous pocket made at the posterior neck. Subsequently, the ECBA module was fixed using bone screws and fibrin glue on the skull where the subdural strip (grid; SDG) was inserted underneath the right hemisphere (Fig. 1c). After filling the burr holes with medical bone cement and subsequent closure of the surgical incision, the location of the ECBA internal module and SDG electrodes was confirmed using fluoroscopic C-arm X-ray imaging (Fig. 1d–f). Postoperatively, CSF leakage or significant complications were not observed except in one dog, which expired five days after surgery due to postoperative infection and intractable seizures.

In this prospective study, all investigations were approved by the Institutional Animal Care and Use Committee at Samsung Medical Center in accordance with the recommendations for handling laboratory animals for biomedical research.

### Experimental protocol of brain stimulation with ECBA during EcoG recording

After a 2-week recovery period, the dogs were re-intubated with an endotracheal tube and anesthetized with isoflurane (1.5–2%) in the prone position. After a careful incision of approximately 2 cm on the neck, the cable tips were pulled out and cleaned thoroughly before connecting to the EEG machine. To minimize the suppressing of brain electrical activities from inhaling the anesthetic agent (isoflurane) and maintain consistent EcoG recording, continuous infusion of intravenous dexmedetomidine (0.3–0.7 μg/kg/hr) was combined with weak general anesthesia (maintained with 0.4–0.8% isoflurane); neither affected spontaneous interictal epileptiform activity nor stimulated motor activity and did not interfere with ECoG recording^18–20^. EcoG was prospectively collected with 512 Hz sampling rates without using a filter in the Nicolet ambulatory EEG system (Natus Medical Inc, San Carlos, CA, USA).

**Figure 2:**
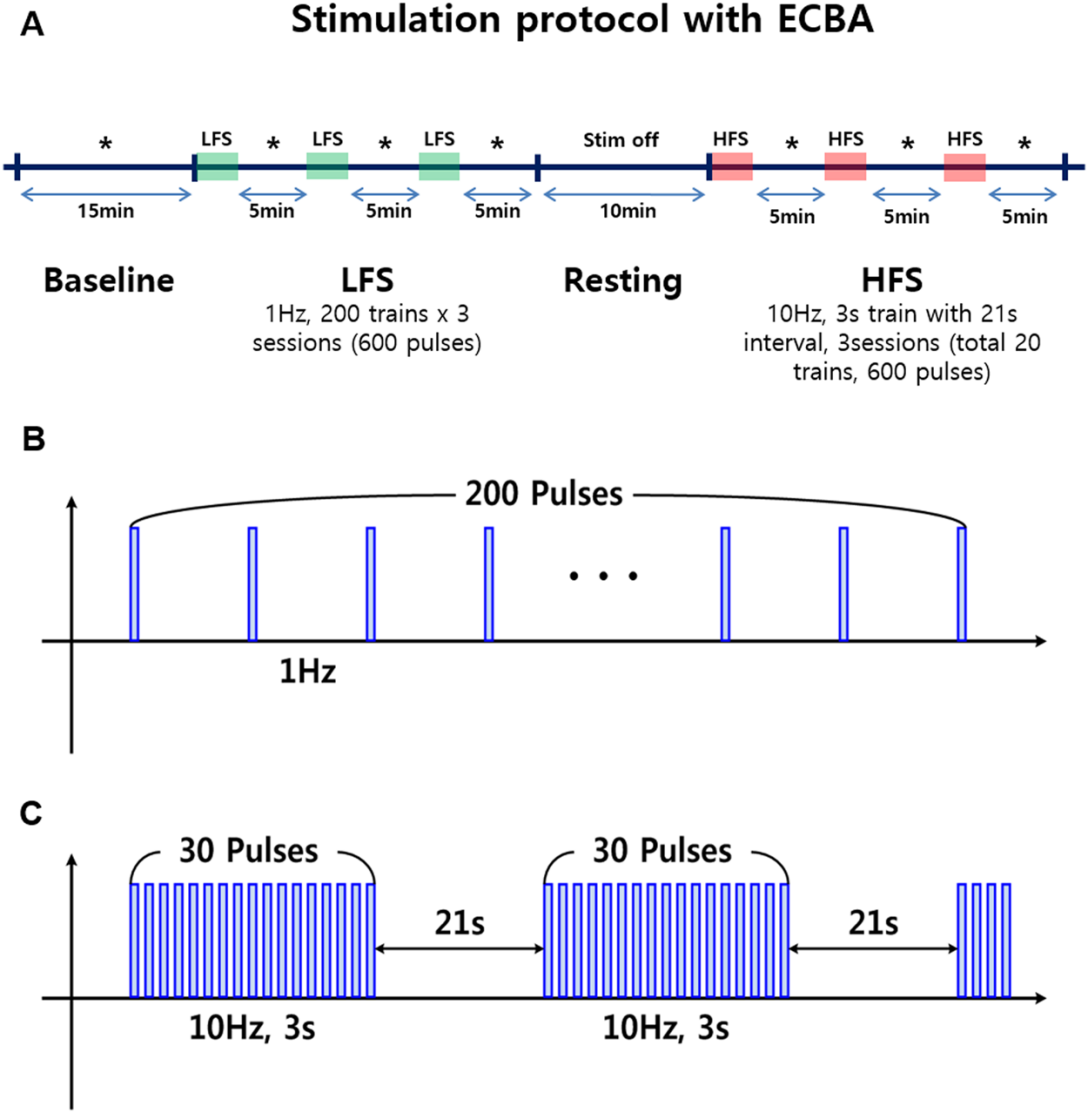
Schematic representation of the stimulation protocol with ECBA. (**A**) Asterisk (*) represents the 15-min EcoG recording period at baseline and immediately after LFS (**B**) and HFS (**C**). ECBA, wirelessly-powered extracranial brain activator; ECoG, electrocorticography; HFS, high-frequency stimulation; LFS, low-frequency stimulation.

Two types of stimulation protocols were used, low-frequency stimulation (LFS) and high-frequency stimulation (HFS), during a simultaneous EcoG recording. After baseline recording for 15 min, three sessions of 200 pulses (1 Hz, 1 mA, 0.5-msec pulse width bi-phasic, charge-balanced, and rectangular waveform, 600 pulses in total) were delivered during the LFS phase, with a 5 min off-stimulation interleaved between sessions. Next, a 15-min resting period was followed by HFS sessions composed of the same LFS pulse shape, 3-sec trains of 10 Hz (30 pulses) with a 21-sec interval. Three sessions of HFS (composed of 7, 7, and 6 trains, total 20 trains and 600 pulses) were performed with a 5-min break interleaved between sessions (Fig. 2). Lastly, to estimate the electrical field difference between transcutaneous and subcutaneous stimulation, the ECBA module was removed from the skull of beagle #2 and placed on the same site just above the skin after a compact suture of the wound. For the transcutaneous stimulation, a wet sponge (approximately 1.4 cm^2^ in size) was attached to the original ECBA electrodes to ensure more even current distribution and the same HFS protocol was applied during 5-min recording.

### Preprocessing and spectral analysis of EcoG data

The 16 channels of ECoG data were acquired at a sampling rate of 512 Hz, and a separate reference electrode was placed between the parietal scalp and posterior neck of the dogs. The data recorded from noisy electrodes were eliminated from the present analysis, and the frequency range of the band-pass filter was adjusted from 0.5 to 110 Hz, including a 60 Hz notch filter. The threshold value of 1 mV was used to determine whether the observed potential value was noise or not^21^.

The spectral power in the data chunk that was divided into 1-sec intervals was estimated using 512-point fast Fourier transform (FFT) without overlap in five frequency bands: 0.5–4 Hz (delta), 4–8 Hz (theta), 8–13 Hz (alpha), 13–30 Hz (beta), and 30–100 Hz (gamma). Then, the log-transformed power spectra in each band of each stage (15-min span of baseline, post-LFS session, and post-HFS session) were calculated to monitor the oscillatory changes generated by the ECBA using a MATLAB analysis tool. Finally, to analyze the EcoG power gradient over intrahemispheric and interhemispheric channels and the stimulator effect located in the right hemisphere, a 3-D model of the beagle brain was used with SPM12 illustrated on surface rendering of mean extracted GM images with MATLAB (MathWorks Inc.) using the Iso2Mesh toolbox^22^.

### Statistical method

To verify statistically significant power spectral changes in the above features estimated from data chunks on the ECoG electrodes among the stages, the independent *t*-test (two-sided) was performed. At the individual patient level, the resulting p-values were corrected for multiple comparisons within all electrodes [false discovery rate (FDR), p < 0.05 or <0.01].

In order to elucidate the stimulation effect as a group level at each stage, we chosed the power spectra of all SDG electrodes in each subject for comparison across the five frequency bands (p pr0.05 or 0.01, uncorrected).

## Results

### Instrument overview

**Figure 3:**
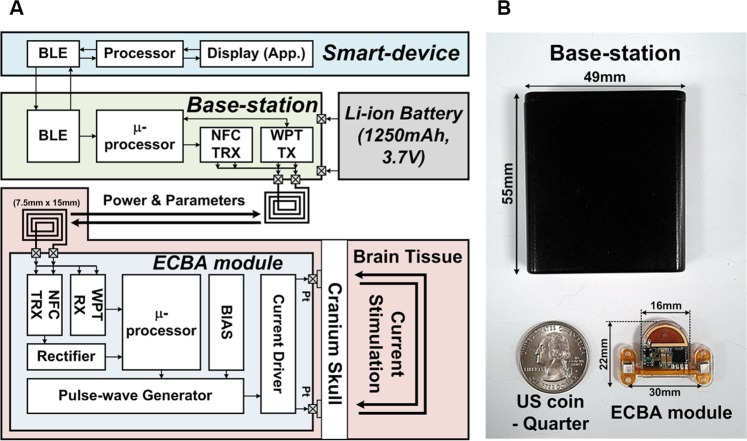
(**A**) Overall architecture of the ECBA system, (**B**) The ECBA external base station (top) and internal module (bottom). ECBA, wirelessly-powered extracranial brain activator.

Figure 3A shows the overall architecture of the ECBA system. The system is composed of an ECBA module, a base station, and a smart device. The module is implanted under the target’s head skin without a battery in the module. The small module injects electrical stimulations to the brain through the skull, corresponding to stimulation parameters provided from the base station. Both power and the control parameters are transferred from the base station to the module and set by specified application of the user’s smart device with NFC communication. The user can connect the smart device to the base station using Bluetooth and monitor the stimulation status with the display.

Figure 3B shows the prototype of the ECBA system. The ECBA module that is 3.3 cm^3^ in size and waterproofed with silicone is securely implanted under the skin. The base station provides power and adjustable stimulation parameters to the module, and the size and weight of the base station is 40.4 cm^3^ and 55 g, respectively, including the rechargeable Li-ion battery. The power transmitted from the base-station to the ECBA module is 0.4 W. The size of a transmitting and receiving antenna is 46 mm × 46 mm and 7.5 mm × 15 mm, respectively. The average power consumption of the ECBA module is 9.62 mW and more than 52.17 mW is required as a peak for the normal operation.

**Figure 4:**
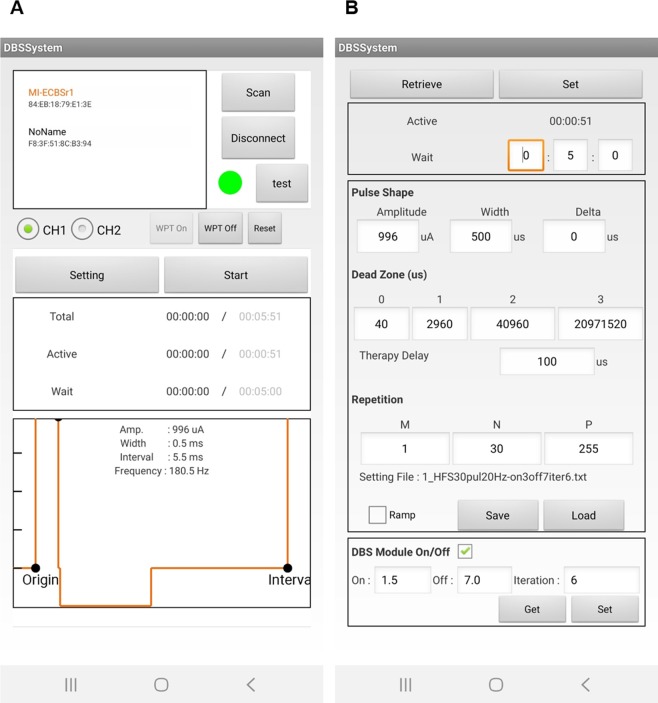
An illustration of the specified application of the smart device for the user interface. (**A**) the main window which the user can check a stimulation status and (**B**) the setting window which the user can adjust parameters.

Lastly, through the user interface at the smart device (e.g. cell phone), wireless power transfer (WPT) function can be controlled on/off at any time (Fig. 4). A user can scan accessible base-stations and choose one for a connection. If a user clicks the setting button, the window is changed to the setting window as shown in Fig. 4B. In the setting window, the user can adjust stimulation parameters; 1) an amplitude and a pulse-width of each phase, 2) intervals of each phase, pulse, train, and cluster and 3) the number of pulses, trains, and clusters. The user can send and write parameters to the module with set button and verifying present parameters with retrieve button. Finally, the user can start the therapy and it can also be stopped with stop button (toggling with start) or WPT off button.

### Voltage difference between transcutaneous and subcutaneous stimulation with ECBA

Compared to transcutaneous stimulation with ECBA, subcutaneous stimulation induced a higher peak-to-peak voltage (4.4 mV *vs*. 0.08 mV, increased amplitude ˃56-fold) during HFS, measured at channel 3 in beagle #2 (Supplementary Figure). Although this finding is based on a peak-to-peak voltage induced at the subdural electrodes by HFS rather than an elaborate electrical field measurement, it represents a large shunt which can diffuse the applied current and result in a substantial loss between scalp and skull.

### EcoG power spectral changes after stimulation in the anesthetized beagle

**Figure 5:**
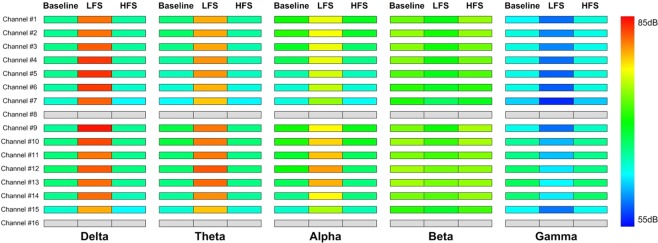
An illustration of EcoG power spectral changes in a beagle (#1) at baseline and immediately after LFS and HFS. A total of 16 channels were placed on the hemisphere ipsilateral (#9–16) or contralateral (#1–8) to the ECBA stimulation site (right hemisphere). ECoG, electrocorticography; LFS, low-frequency stimulation; HFS, high-frequency stimulation; ECBA, wirelessly-powered extracranial brain activator.

The analysis of power spectra revealed a consistent and prominent modulation of the spontaneous oscillatory activity evoked by ECBA. In the low-frequency band (delta, theta, and alpha) each beagle showed a clear increase in absolute power across ipsilateral and contralateral EcoG channels after LFS compared to baseline (Fig. 5). By contrast, a decrease in beta and gamma power was evident across all channels with LFS. Similarly, HFS resulted in a significant decrease of power in the delta, theta, and alpha bands and a distinct increase in beta and gamma power at nearly all channels over the hemisphere ipsilateral (#1–7) or contralateral (#9–15) to the ECBA (right hemisphere) stimulation site.

### Topographic EcoG spectral changes on individual electrodes induced by ECBA

**Figure 6:**
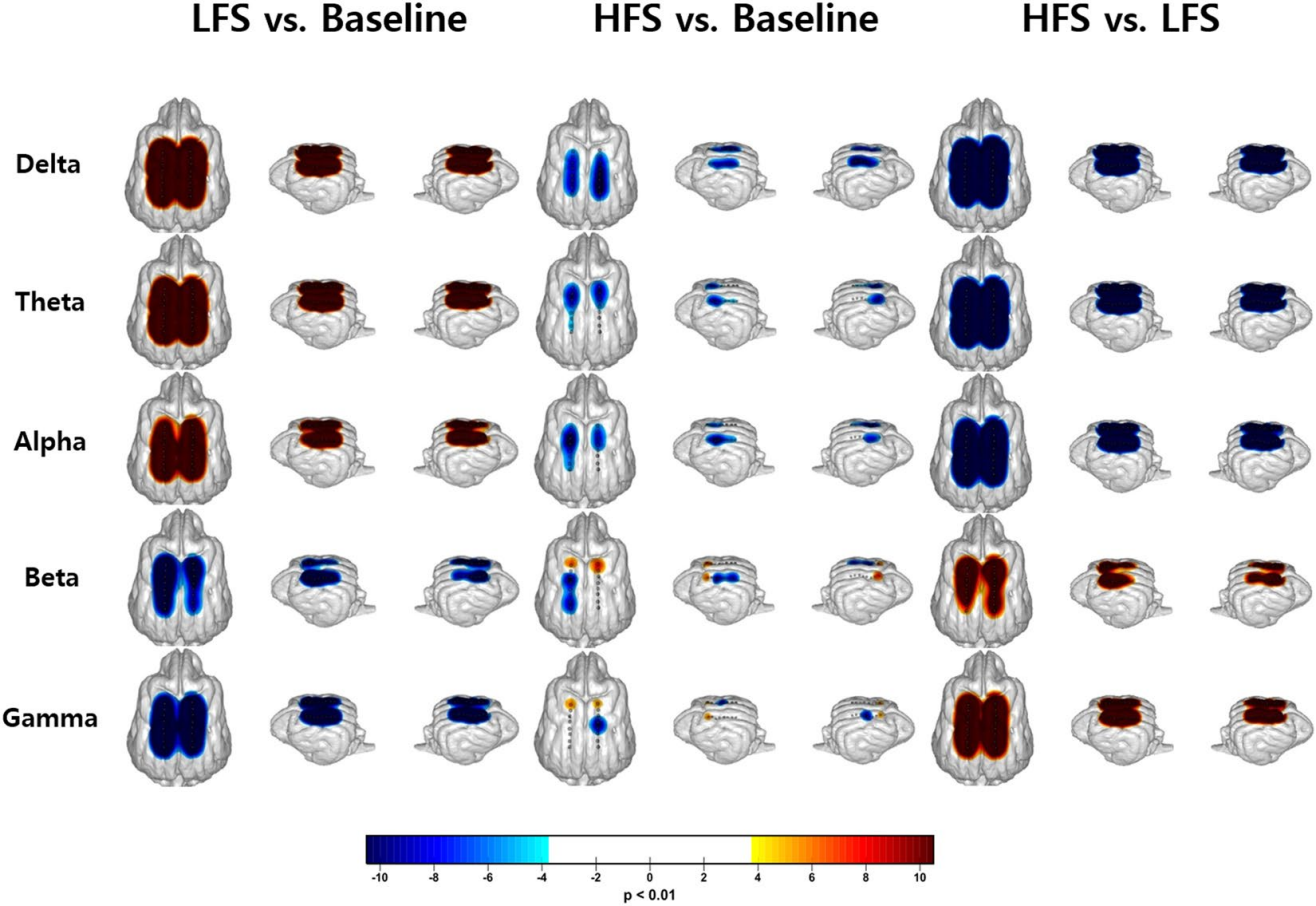
Topographic pattern of relative EcoG power spectral changes from LFS and HFS induced by ECBA with a 3-D brain modeling of subdural strip electrodes. The figure indicates the significant changes between baseline, LFS, and HFS (p an0.01, FDR corrected). EcoG, electrocorticography; LFS, low-frequency stimulation; HFS, high-frequency stimulation; ECBA, wirelessly-powered extracranial brain activator; FDR, false discovery rate.

Using a 3D brain modeling of subdural strip electrodes superimposed on the dorsal brain surface, diffuse and prominent power spectral changes (increase in delta, theta, and alpha frequencies; decrease in beta and gamma frequencies) were observed in parallel after LFS, compared to baseline spectral power (p ba0.01, FDR corrected; Fig. 6, left). HFS also induced a similar, but opposite, pattern of power change (decrease in delta to alpha frequency range in both hemispheres and increase in beta to gamma range at both sensory cortices), however, some inconsistent topographic patterns were observed at the cortex on the stimulation side in gamma frequency and the contralateral cortex in beta frequency (increase at the anterior sensory cortex, but decrease in middle to posterior lateral cortex of the parietal lobe; Fig. 6, middle). Compared to the power change after LFS, HFS induced similar, but more consistent, spectral power changes (decrease in delta, theta, and alpha frequencies but increase in beta and gamma frequencies; Fig. 6, right).

### Group analysis of EcoG modulation induced by ECBA

**Figure 7:**
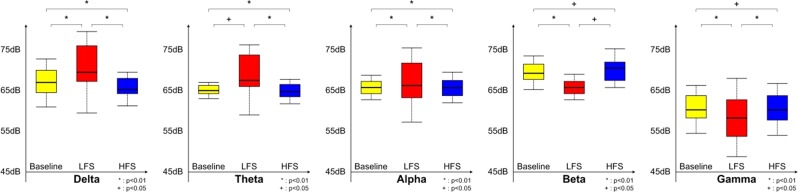
Group data of EcoG power spectral density at each stage (baseline, LFS, and HFS) measured in four beagles during the experiment. Results show a significant LFS-induced facilitation at low frequency band and HFS-induced augmentation of high frequency rhythm compared with baseline across all channels (p sh0.01 or 0.05). Conversely, LFS and HFS resulted in an attenuation of high frequency and low frequency rhythms, respectively (p  r0.01 or 0.05). EcoG, electrocorticography; LFS, low-frequency stimulation; HFS, high-frequency stimulation.

At a group level, LFS-related oscillatory activity also facilitated delta or alpha (p < 0.01) and theta power (p < 0.05), whereas HFS induced an attenuation of low frequency power (p < 0.01) compared to baseline across all channels. Similarly, high frequency (beta and gamma) band power was significantly decreased by LFS (p < 0.01) and increased after HFS (p < 0.05) compared to baseline. Similar results were observed between oscillatory changes after LFS compared to after HFS (Fig. 7).

## Discussion

In the present study, development of the ECBA was described for subcutaneous, extracranial neurostimulation system for neuromodulatory therapy in beagles, which may ultimately be applied to humans with intractable neurological disorders. The ECBA is wireless technology supporting a tES with freely adjustable stimulation modes. The *in vivo* proof-of-principle tests demonstrated the ECBA compared favorably to an established tES system which is widely used in basic and clinical research.

A significant voltage difference was found between transcutaneous and subcutaneous skin effects with HFS, which apparently was several times higher with subcutaneous HFS considering the different electrode size in the two conditions. In a recent study, the 3-D spread of electric fields was quantified in both rodents and human cadavers, showing the scalp and adjacent soft tissues function as an effective shunt, resulting in at least approximately 80% loss of applied current^17^, which was very similar to the results in the present study. In previous studies^23–25^, a 1 mV/mm voltage gradient was reportedly required to consistently affect neuronal firing and phase entrain brain rhythms in response to arbitrary stimulus frequencies. Because currents larger than 2 mA are usually avoided due to uncomfortable skin sensations, phosphenes, and other side effects^26,27^, a current greater than 4–6 mA to achieve 1 mV/mm in brain tissue^17^ must be over-stimulating and may be dangerous for long-term application to animals or in clinical research. Therefore, ECBA is a good alternative precluding a suboptimal neurostimulation which may result in uncertain therapeutic effects.

In addition to the energy-efficient and freely-adjustable neurostimulation features, ECBA has significant advantages such as wireless power supply, battery-free, and possibility of further miniaturization for subcutaneous implantation allowing a variety of neuromodulation therapies. A new version of the device using waterproof, titanium, and polymer packaging materials that showed satisfactory results after more than half a year of reliable implantation and long-term *in-vivo* testing has been designed by our research team.

Electric fields induced non-invasively through the scalp (tES) may probe neural oscillatory or connective traits and potentially ameliorate brain disease^23,28,29^. The effects of tACS depend on the involved parameters such as current intensity, frequency, and size and location of the electrodes. However, the physiological mechanisms of tES remain elusive.

Many studies have been conducted on the physiological mechanism of tACS algorithms of 10 Hz for the facilitation of normal, motor, visual, and cognitive functions^30–33^. Antal *et al*.^30^ reported that a tACS at various frequencies (1–45 Hz) was delivered over the left motor cortex of healthy subjects, and only 10 Hz tACS slightly improved implicit motor learning but did not alter cortical excitability. A study of visual processing with tACS showed that in a light condition, perception of phosphenes was most effective in the beta frequency range (14–20 Hz), whereas in the dark condition, phosphenes were most effectively induced by alpha frequency stimulation (10–12 Hz)^32^. Moreover, 10 Hz tACS over frontal^33^ or parieto-occipital areas^31^ augmented not only individual creative subscales such as originality and elaboration, but also abstractness of titles, and posterior alpha activity which outlasted the stimulation relative to sham stimulation.

Studies comparing low-frequency tACS (around 1 Hz) to HFS have seldom been conducted. Recently, a large amplitude rhythmic slow oscillation (SO; approximately 1 Hz) was introduced and shown to entrain electrical activity at cortical and hippocampal sites^34,35^. SO activity has also been successfully entrained using rTMS^36^ or voltage sensitive dye imaging^37^, which decreased cortical power and cortico-cortical coherence compared with baseline awake or the anesthetic-induced sleep state^38^. Direct comparison of the ECBA system in the present study with previous study results may be difficult due to the divergent stimulation parameters used: current intensity and frequency, rectangular pulse shape rather than sinusoidal waves, and different anesthetic agents.

Because specific frequencies of brain oscillations reflect particular ongoing cognitive or sensory-motor processes^39,40^, tACS may augment or attenuate ongoing processes through exogenous intervention of those oscillations^41^. Therefore, tACS has a role in modulating frequency-specific neuronal networks, which leads to cognitive or behavioral changes. However, the beneficial effects of tACS in clinical fields^42–44^ have been reported in only a few studies and less is known regarding the mechanisms and function reorganization from tACS. Therefore, more studies are necessary to discover not only the synaptic mechanisms involved but optimal stimulation parameters for use in basic research and clinical practice. tACS has the potential to become a new therapeutic modality for brain stimulation with a very low risk of adverse effects.

In the present study, dexmedetomidine (DEX) was chosen as the main anesthetic agent for sedation during the experimental sessions. DEX alters arousal primarily through actions on presynaptic α2 adrenergic receptors on neurons projecting from the locus coeruleus. Binding of DEX to the α2 receptors hyperpolarizes locus coeruleus neurons, which creates a loss of inhibitory inputs to the preoptic area of the hypothalamus and activates these inhibitory pathways from the preoptic area to the arousal centers^45,46^. A low-dose infusion of DEX induces an EEG change showing a combination of slow delta oscillations with spindles^47,48^, which closely resemble the spindles that define stage II non-REM sleep^47^. The synergistic effect of isoflurane combined with DEX resulted in a consistent^49^ but arousable sedation through endogenous sleep pathways^46^. Moreover, in a pharmacokinetic study^50^, a steady-state low-dose serum DEX concentration was achieved through a constant-rate infusion of DEX administered for several hours at the same dosage without any cumulative effects. Therefore, in the present study, the experiment could reliably proceed under a constant DEX infusion with close monitoring of sleep spindles. DEX may act as a biomarker to prevent or minimize the loss of valuable synaptic or network information induced by tACS from excessive deep anesthesia, such as a high concentration of isoflurane.

In the present study, we measured peak-to-peak voltages of the subdural electrodes with respect to the reference to quantify the electrical shunting effect of the skin. A limitation of this measurement, however, is that we used two different configurations for the electrode-to-tissue contact, and consequently the results included the shunting effect as well as difference of the electrode configuration. Thus, as a next step to validate the need of the subcutaneous device, we have just started a study focused on assessment of electric field penetration via subcutaneous and transcutaneous electrical stimulation. In addition, we plan to combine functional neuroimaging methods (including functional MRI or functional near-infrared spectroscopy) and record the brain oscillatory changes from multiple sites (e.g., stereoEEG method) to investigate the immediate and after-effects of ECBA at the whole brain level.

In conclusion, The ECBA is a small, battery-free, and freely-adjustable neurostimulation system allowing long-term *in vivo* recording. Most importantly, the ECBA exhibited less susceptibility to current passing the scalp before reaching the cortex and effective modulation of neural activity measured by EcoG recording. Taken together, these results demonstrate the utility of the ECBA as an effective, subcutaneously implanted, safe, and long-term non-invasive neuromodulation device for treatment of human brain disorders.

## Supplementary information

Supplementary figure
