# Supplementary material for: Development of wirelessly-powered, extracranial brain activator (ECBA) in a large animal model for the future non-invasive human neuromodulation
Source: Sci Rep. 2019 Jul 29;9:10906. doi: 10.1038/s41598-019-47383-2 (PMC6662771; doi:10.1038/s41598-019-47383-2)
Supplement: Supplementary file 1 — Supplementary figure [file 41598_2019_47383_MOESM1_ESM.pdf]

Development of wirelessly-powered, extracranial brain activator (ECBA) in a large animal model for the future non-invasive human neuromodulation

Hyungwoo Lee[1,†], Jin San Lee[2,†], Yeongu Chung[3], Woo Ram Chung[4], Sang Joon Kim[1], Joon Seong Kang[1], Sung Min Park[5], Wonok Kang[5], Dae Won Seo[4], Duk L. Na[4], Young-Min Shon*[,4]

Supplementary figure

Comparison between the effect of transcutaneous and subcutaneous stimulation with ECBA from a beagle model. For the transcutaneous stimulation with ECBA, we used two saline-soaked sponges attached on the electrodes of ECBA. The peak-to-peak voltage change after the subcutaneous stimulation with ECBA was 4.4 mV, which was 56 times larger than that after the transcutaneous stimulation (0.08 mV).

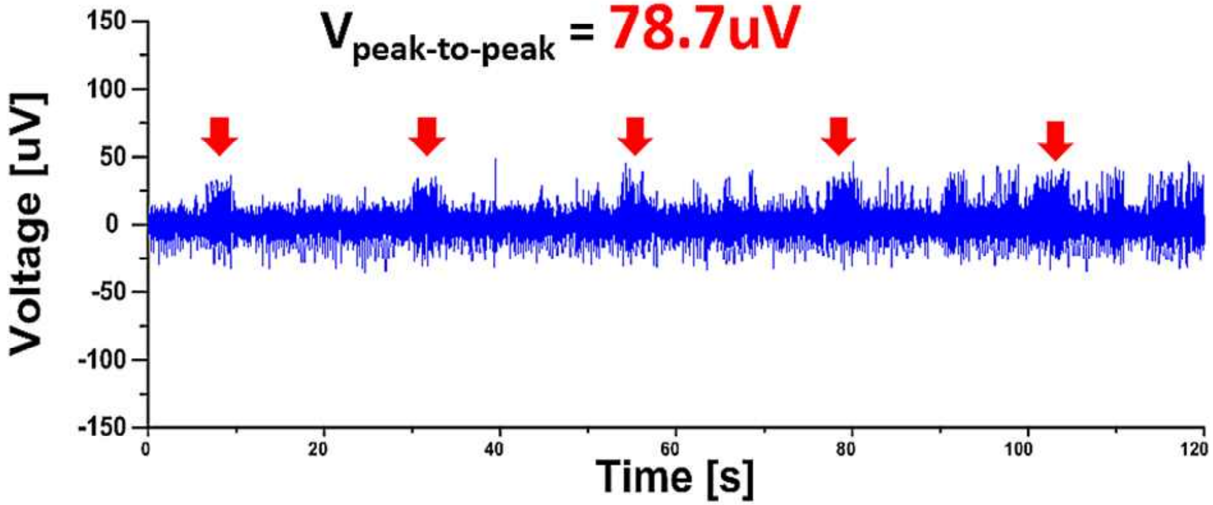

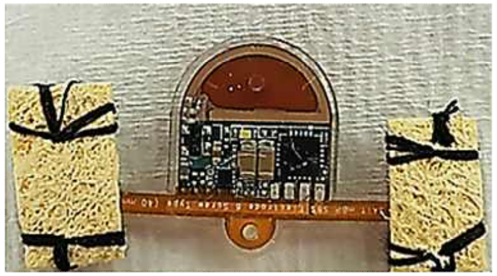

Transcutanous HFS
with ECBA
(Saline-soaked sponge
attached to electrodes)

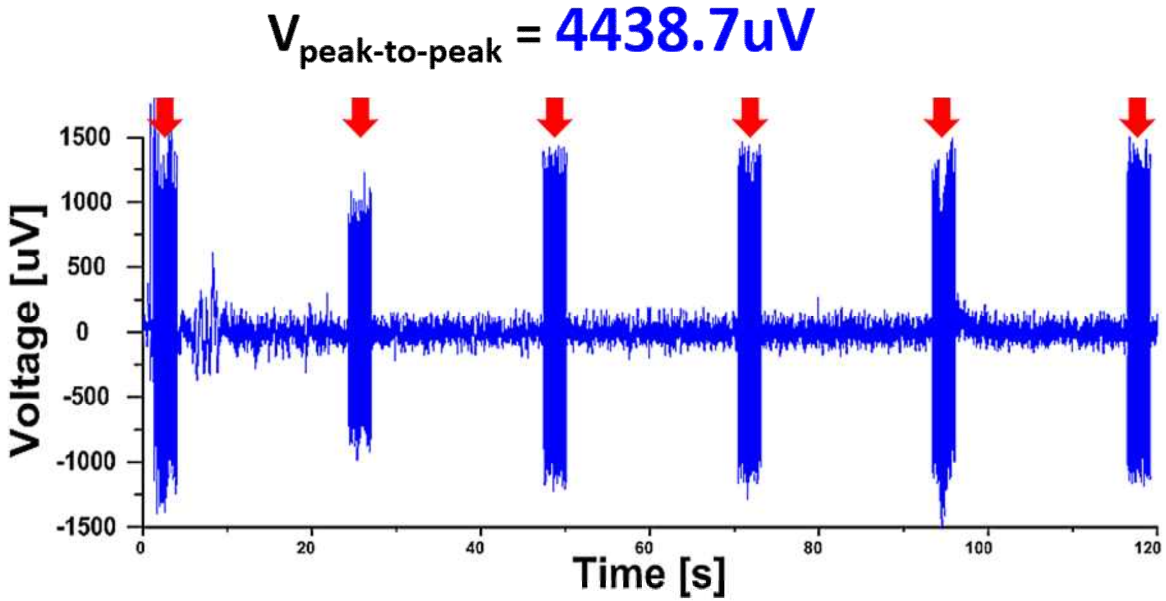

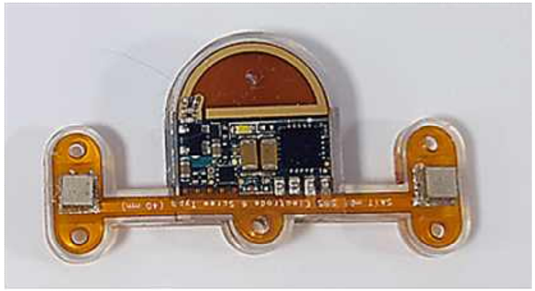

Subcutanous HFS
with ECBA
